# A new high-resolution global topographic factor dataset calculated based on SRTM

**DOI:** 10.1038/s41597-024-02917-w

**Published:** 2024-01-20

**Authors:** Yuwei Sun, Hongming Zhang, Qinke Yang, Rui Li, Baoyuan Liu, Xining Zhao, Haijing Shi, Hongyi Li, Yuhan Ren, Xiao Fan, Liang Dong, Yikun Xu, Yi Chang, Linlin Yuan

**Affiliations:** 1 College of Information Engineering, Northwest A & F University, Shaanxi, 712100 China; 2 Agricultural Information Intelligent Sensing and Analysis Engineering Technology Research Center, Shaanxi, China; 3 Key Laboratory of Agricultural Internet of Things, Ministry of Agriculture and Rural Affairs, Shaanxi, China; 4 Department of Urbanology and Resource Science, Northwest University, Shaanxi, 710069 China; 5 Institute of Soil and Water Conservation, Northwest A&F University, Yangling, 712100 China; 6 Advanced Institute of Natural Sciences, Beijing Normal University, Zhuhai, 3621086 China

**Keywords:** Geomorphology, Environmental impact

## Abstract

Topography is an important factor affecting soil erosion and is measured as a combination of the slope length and slope steepness (LS-factor) in erosion models, like the Chinese Soil Loss Equation. However, global high-resolution LS-factor datasets have rarely been published. Challenges arise when attempting to extract the LS-factor on a global scale. Furthermore, existing LS-factor estimation methods necessitate projecting data from a spherical trapezoidal grid to a planar rectangle, resulting in grid size errors and high time complexity. Here, we present a global 1-arcsec resolution LS-factor dataset (DS-LS-GS1) with an improved method for estimating the LS-factor without projection conversion (LS-WPC), and we integrate it into a software tool (LS-TOOL). Validation of the Himmelblau–Orlandini mathematical surface shows that errors are less than 1%. We assess the LS-WPC method on 20 regions encompassing 5 landform types, and R^2^ of LS-factor are 0.82, 0.82, 0.83, 0.83, and 0.84. Moreover, the computational efficiency can be enhanced by up to 25.52%. DS-LS-GS1 can be used as high-quality input data for global soil erosion assessment.

## Background & Summary

Soil erosion is a global hazard, as it exerts serious and negative impacts on ecosystem services, crop production, drinking water, and carbon stocks^1–3^. Recent studies have revealed that global soil erosion has more severely increased due to population growth, economic development, and climate change^4,5^. Researchers, governments, policy-makers, and conservation organizations worldwide are confronted with the challenge of devising innovative strategies to alleviate the pressures due to accelerated soil erosion^6,7^. The Universal Soil Loss Equation (USLE)^8^, the revised version (RUSLE), and the Chinese Soil Loss Equation (CSLE)^9^ have gained wide usage for estimating the soil erosion risk owing to their simplicity and robustness. Nonetheless, the acquisition of a substantial amount of model input data is a significant challenge in terms of both space and time, particularly concerning topography^10,11^, typically represented in models as a combination of the slope length and steepness (LS-factor). Furthermore, the processing of data from different sources at multiple scales is an exceedingly time-consuming and error-prone task, resulting in a significant portion of the research time dedicated to data preparation rather than the application and analysis of soil erosion modelling. Unfortunately, neither a global seamless high-resolution LS-factor dataset nor an efficient method for extracting LS-factor on a global scale is yet available.

The LS-factor can be acquired from digital elevation models (DEMs) at regional scales^12,13^, which can be obtained through ground surveys, existing topographic maps, or remote sensing images^14,15^. With technological advances, remote sensing platforms (satellites, space shuttles, etc.) are increasingly used to acquire high-quality surface elevation data^16,17^, ranging from localized super high-resolution DEMs (i.e., LiDAR DEMs) to high-resolution global DEMs (GDEMs)^18^. Although LiDAR DEMs are of high accuracy, they are limited to relatively few countries due to the prohibitive cost, accounting for approximately 0.005% of the Earth’s land area^19^. Consequently, spaceborne GDEMs generated from radar and optical sensors constitute the primary source of elevation information for the majority of global regions^18^, offering resolutions up to 1-arcsec (approximately 30 m at the equator). Considering the limited penetration of radar signals in dense vegetation, it is crucial to recognize that, strictly speaking, all GDEMs function as global digital surface models (GDSMs)^20^, and they do not accurately represent bare ground elevation in vegetated regions^21–23^. Notably, the slope values were largely unaffected while correcting for the elevation values^24^. In contrast, calculations of the slope length, defined as the horizontal distance from the starting point along the vertical contour line to the slope deposit or obvious channel^25^, are independent of the vertical height. Instead, the resolution of the DEM becomes a critical factor influencing slope length values, often more so than DEM sources^26^. Therefore, when calculating the topographic factor, GDSMs are treated as equivalent to GDEMs. For simplicity, we use the term DEM in the rest of this paper.

Several GDEM products, including the 1-arcsec Advanced Spaceborne Thermal Emission and Reflection Radiometer (ASTER)^27^, the Shuttle Radar Topography Mission (SRTM)^28^, and the 3-arcsec Multi-Error-Removed Improved-Terrain (MERIT) DEM^29^, have become freely accessible to the public since 2000. In previous studies, it has been indicated that in regard to slope steepness and slope length values directly dependent on grid size calculations, finer-resolution datasets are superior to coarse-resolution ones^24,30^. Among the various 1-arcsec GDEM products, the SRTM is one of the most successful GDEMs despite the presence of voids and nonnegligible vertical errors^19,31,32^. The most recent additions to the family of 1-arcsec GDEMs, such as the global Advance Land-Observing Satellite (ALOS) world 3D-30 m (AW3D30) DEM and the Copernicus DEM, could likely provide better performance levels due to the improved processing techniques and the inclusion of more data. Validation of these new products over areas with variable topographical and land cover conditions is limited due to the short availability period. With the utilization of enhanced processing techniques and multisource data fusion^33^, preliminary SRTM products have been consistently refined, with notable instances, including voids in SRTM version 3.0 (V3) GDEM^34^, effectively corrected, and the absolute vertical accuracy greatly exceeds the 16-m accuracy requirement in the original SRTM specification^35^.

The availability of increasingly high-quality SRTM products has greatly influenced global soil erosion assessments. A notable example is the release of the Global Soil Erosion Modelling Platform (GloSEM) dataset in 2019^1^, providing a comprehensive evaluation of global soil erosion over an area of 125 million square kilometres (approximately 84% of the Earth’s surface). The LS-factor is typically calculated based on SRTM 3-arcsec spatial resolution data to represent the effect of topography on soil erosion, and a resampled LS-factor input layer at a 25-km resolution has been provided. Furthermore, the GloSEM 1.3 dataset^36^, launched in 2022, specifically focuses on assessing global soil erosion in croplands, covering an area of 1.4 billion hectares (approximately 10% of the global land surface). The LS-factor has been calculated using hole-filled SRTM and ASTER GDEM v2 data with a 3-arcsec spatial resolution. Ultimately, the local combination layer of climate, soil, topography, farming, and management system at 100 m is needed. Despite the significant endeavours to acquire global soil erosion data, it is important to acknowledge that existing datasets still possess limitations. The challenge of achieving a global high-resolution and high-precision LS-factor dataset (DS-LS-GS1) remains unresolved, impeding comprehensive global soil erosion assessments.

However, due to the unique characteristics of SRTM data and the large-scale aspect of the application process, a new algorithm must be developed specifically for the DS-LS-GS1 project. Conventional algorithms typically employ grids in the projected coordinate system for extracting the slope length and slope steepness^37^, whereas SRTM data utilize latitude-longitude (geographic) grids. The projection transformation process involves mapping spatial geodetic coordinates onto a plane through mathematical transformations in a plane rectangular coordinate system. Although researchers have often overlooked the error caused by projection at the watershed scale, its impact becomes significant when considering the global scale, with bias variations according to the latitude and projection scheme^38^. Several case studies have been conducted to extensively evaluate the differences that arise when transitioning from geographic to projected coordinate systems. For instance, when calculating the true area of a large-scale region, biases can emerge in projected grids, and these biases can reach approximately 2% and 4.5% at global and regional scales, respectively^38^. The issue of transforming the grid coordinate system suggests that geographic coordinates should be preferred when calculating the grid cell size (GCS)^39,40^.

In recent years, using geographic coordinate systems, many area-based social and environmental indicators (such as the population density^41^, coastline assessment^42^, and watershed area^43^) have been evaluated based on latitude-longitude grids for more accurate global analysis. However, the development of algorithms for extracting the slope length and slope steepness from latitude-longitude grids on a global scale remains unresolved. The calculation process that defines the GCS is crucial because the cell size measurement unit varies in different grid coordinate systems. Furthermore, GCS data serve as fundamental data for the computation of the slope steepness and slope length, particularly the slope length. Notably, the calculation of the slope length is influenced by the cumulative effect of the size of each cell. When using latitude-longitude geogrids, the GCS decreases towards the poles, necessitating recalibration of the size of each grid. Addressing this issue is of paramount importance. Reference change and planimetric projections are critical steps and error and approximation sources^38^. To accurately map the LS-factor on a global scale, the algorithm should be refined to directly estimate the slope length and slope steepness in latitude-longitude grids.

In this study, we present a global-scale and high-resolution (1-arcsecond) LS-factor dataset with an improved method to estimate the LS-factor without projected conversion based on the SRTM (LS-WPC method), which recalculates the value of each grid cell size (GCS) and updates the corresponding slope steepness and slope length computation equations. The LS-WPC method is integrated into a software tool (LS-TOOL), which facilitates the subsequent calculation of the LS-factor. The generic verification approach is to use a DEM defined by mathematical surfaces; thus, the true output value can be predetermined to avoid uncertainty due to uncontrollable data errors^44^. The LS-WPC method is validated against Himmelblau–Orlandini mathematical surfaces (HOMSs) at a resolution of 1 m, as well as against SRTM data across varying topographic conditions. Notably, the coefficient of variation (CV) values of some previously published LS-factor datasets with DS-LS-GS1 reveal suitable agreement. These results provide data support for assessing the global soil erosion risk and comprehensive evaluation of soil health and ecosystem service functions. DS-LS-GS1 provides a basis for identifying potential hotspots and land management across different scales. In addition, this dataset can be considered in the comparison of the LS-factor to other regional- or global-scale studies in the future.

## Methods

### Data preprocessing and quality assessment

#### Data source and preprocessing

**Fig. 1:**
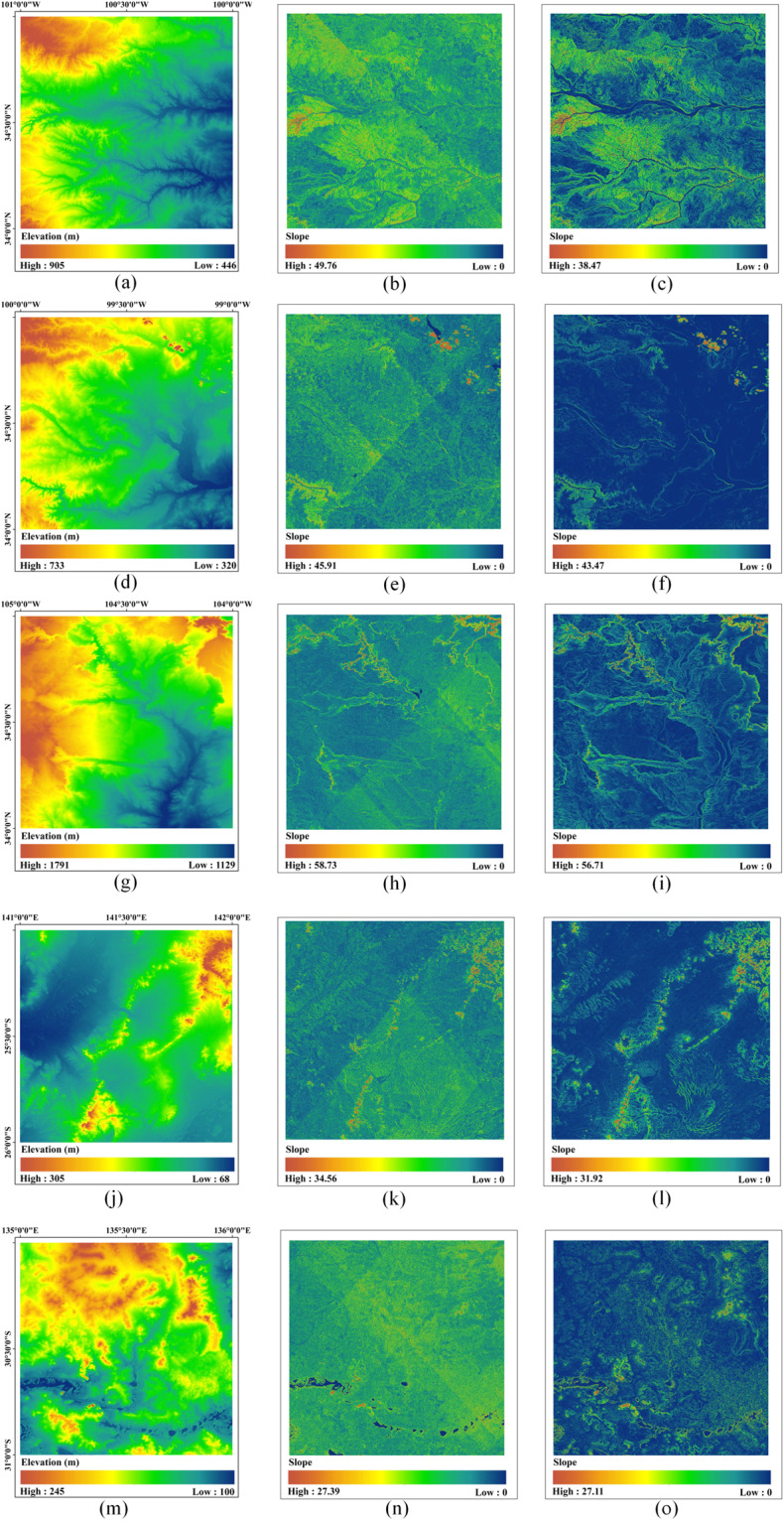
(**a**),(**d**),(**g**),(**j**),(**m**) Original SRTM data; (**b**),(**e**),(**h**),(**k**),(**n**) Slope map calculated based on SRTM; (**c**),(**f**),(**i**),(**l**),(**o**) Slope map calculated based on the denoised SRTM.

As the foundation for all computations, we employed the void-filled SRTM V3 global 1-arcsec product^34^, derived from the reprocessing of SRTM data. This product incorporates enhancements involving the elimination of all voids through filling in ASTER GDEM2, USGS GMTED 2010, and USGS National Elevation Dataset, resulting in an improved vertical accuracy^45^. Despite the significant enhancements in the SRTM quality, notable stripe errors persisted in slope calculations (Fig. 1b,e,h,k,n). To address this issue, we employed a denoising method based on the optimization of a low-rank group-sparse model^46^. This approach effectively mitigated the impact of mixed errors, such as spikes, speckles, and multidirectional stripes while preserving the resolution and topographical structure^47^. As a result, the slope calculation accuracy was significantly improved (Fig. 1c,f,i,l,o), with an impressive error elimination rate of 97.6% relative to the original data.

#### Denoised SRTM (SRTM-D) product details

**Fig. 2:**
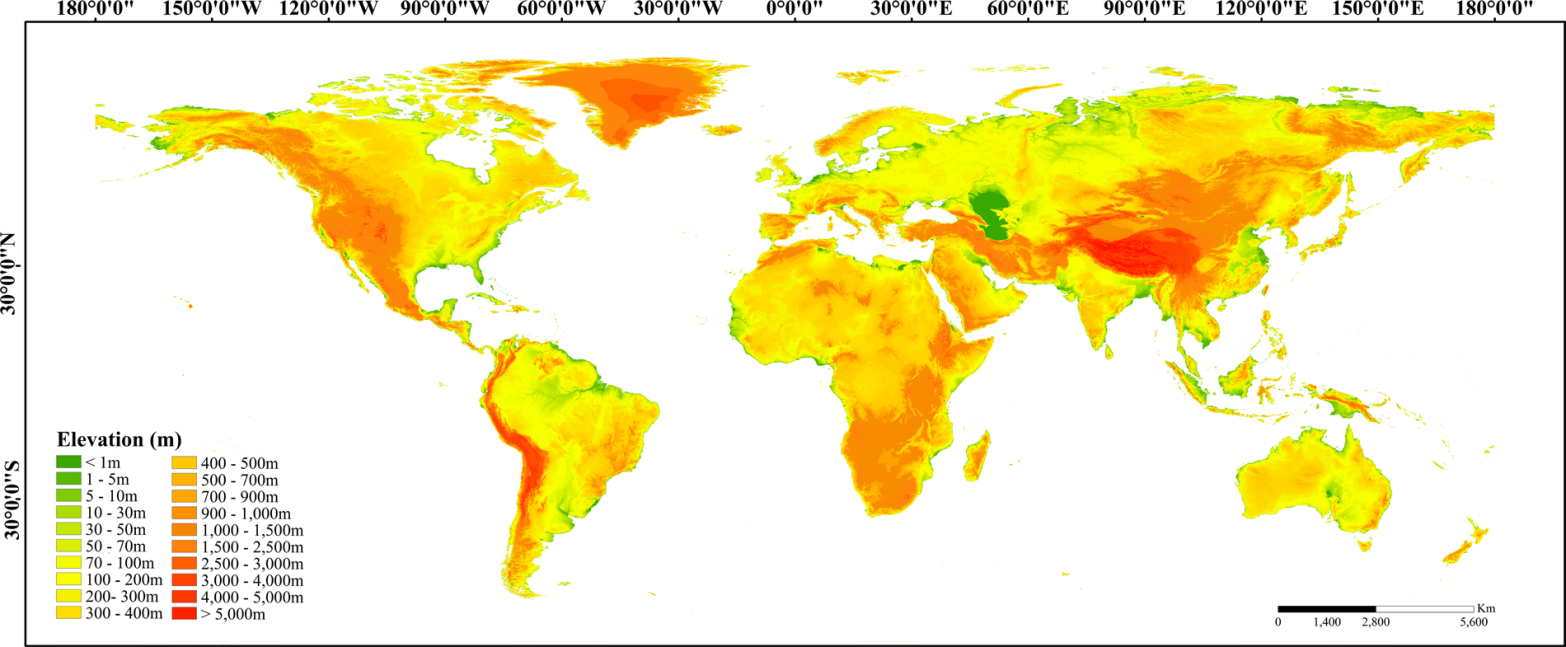
1-arcsecond global elevation data.

The SRTM-D product is divided into 1° x 1° latitude and longitude tiles using geographic projection, horizontally referenced to the World Geodetic System 1984 (WGS84) and vertically to the Earth Gravitational Model 1996 (EGM96)^24^. Geocoded SRTMs can be seamlessly integrated with similar data obtained from other sensors into geographical information systems. Since the SRTM data covering the 60°N to 56°S latitude range only span approximately 80% of the land area, we supplemented it with resampled MERIT DEM^29,48,49^ data, covering the 60°N–83°N range. The final combined dataset, comprising SRTM-D data at a 1-arcsec resolution, spans the land area between 83°N and 56°S (Fig. 2), encompassing over 99% of the global landmass (excluding Antarctica).

#### Comparison of the DEM-derived LS-factor layer quality

In comparing data sources for generating the LS-factor layer, we assessed the sensitivity of the outcomes based on the DEM quality. First, we compared the SRTM-D product with the MERIT DEM, noting that the coarser resolution of the MERIT DEM resulted in blurred terrain details, elongated slope lengths, reduced steepness, and increased LS-factor values (Supplementary Table 1). The preference for the 1-arcsec SRTM-D product over the 3-arcsec MERIT DEM lies in the finer spatial resolution of the former, which is crucial for capturing detailed variations in the LS-factor. Additionally, we evaluated the SRTM-D product against the AW3D and Copernicus datasets. Despite the limited validation of these newer datasets, we verified the accuracy in five target areas with various landscapes. We computed the slope steepness, slope length and LS-factor for the Copernicus, AW3D, and SRTM-D datasets, observing similar calculation errors across all three datasets (Supplementary Table 2). Notably, the SRTM-D dataset slightly outperformed the others in terms of LS-factor calculation errors across the various topographic conditions in most cases. Overall, these analyses collectively underscored the quality of the LS-factor layer derived from the SRTM-D dataset.

### Computational stages

**Fig. 3:**
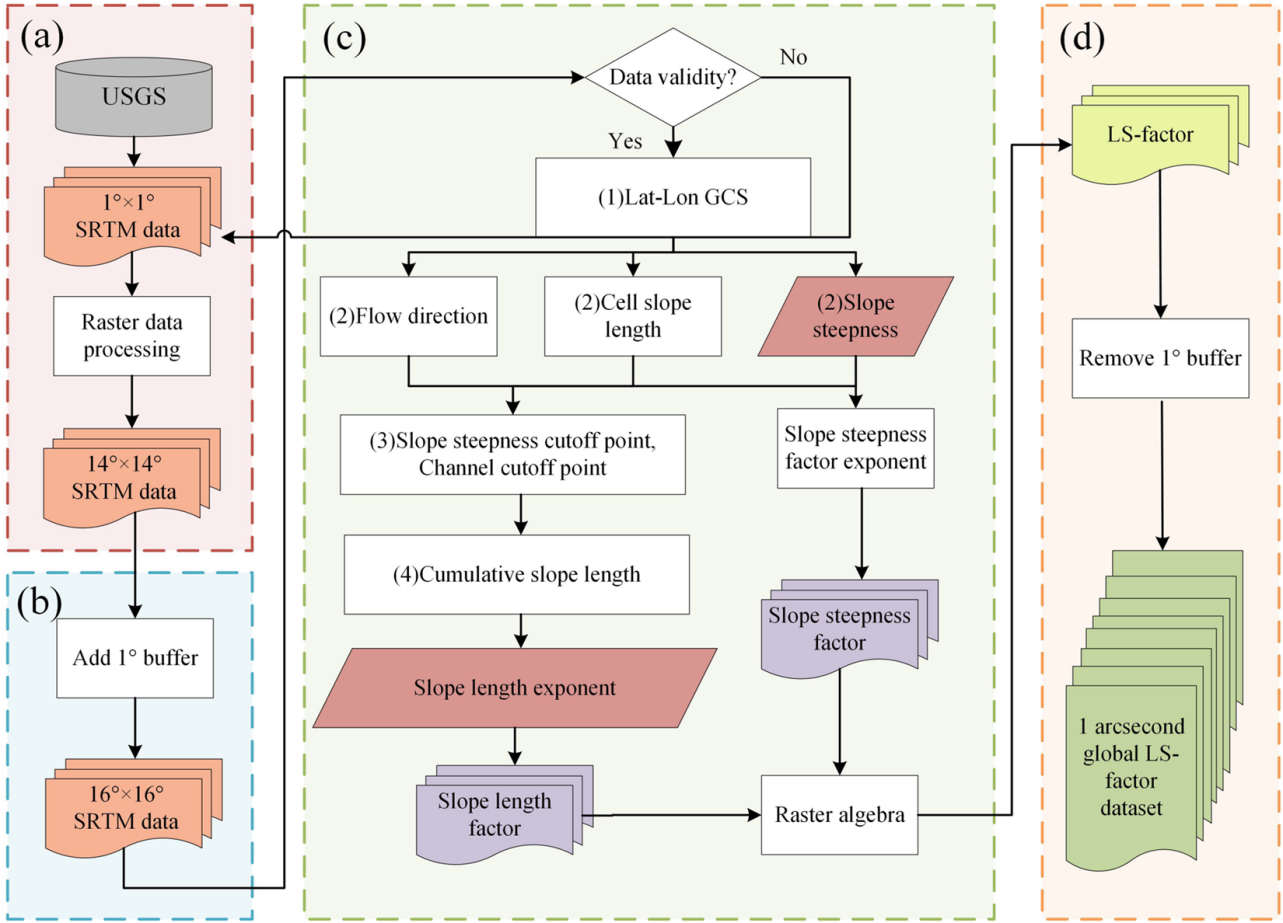
The procedure flowchart describing the production of global LS-factor dataset.

The overall computation of the global LS-factor dataset consisted of the following five steps (Fig. 3):

- Merge the single SRTM-D tiles (1° × 1°) into larger tiles (14° × 14°) to address the high computational demand on a global scale.
- Add a 1° buffer to each SRTM-D tile for preventing edge information loss.
- Compute the slope length, slope steepness, L subfactor, S subfactor, and LS-factor on a global scale.
- Remove the 1° buffer from each SRTM-D tile to generate the global LS-factor dataset.

The specific LS-factor extraction process for global elevation data is shown in Fig. 3. Considering the computational efficiency and the cumulative impact of each cell size on the slope length calculation, the process may extend across thousands or even tens of thousands of grid cells. Therefore, the LS-factor was extracted within 16° × 16° using the LS-WPC method, where the algorithm consists of the following steps: (1) calculation of the grid cell size using latitude and longitude information (Lat-Lon GCS); (2) determination of the flow direction, slope steepness, and cell slope length on the basis of the GCS and single-flow deterministic 8 (D8) algorithm; (3) establishment of the slope steepness cut-off point according to the slope steepness and cut-off factor, where the flow direction is used to calculate the catchment area, and the specified threshold value is used to set the cut-off point of the channel network; (4) calculation of the cumulative slope length by referencing the cut-off position; and (5) computation of the LS-factor using the slope steepness and slope length according to the CSLE. In this process, the input SRTM-D data are ASCII data, and the validity of the input data was assessed before the calculation.

### Merging the single SRTM-D tiles

**Fig. 4:**
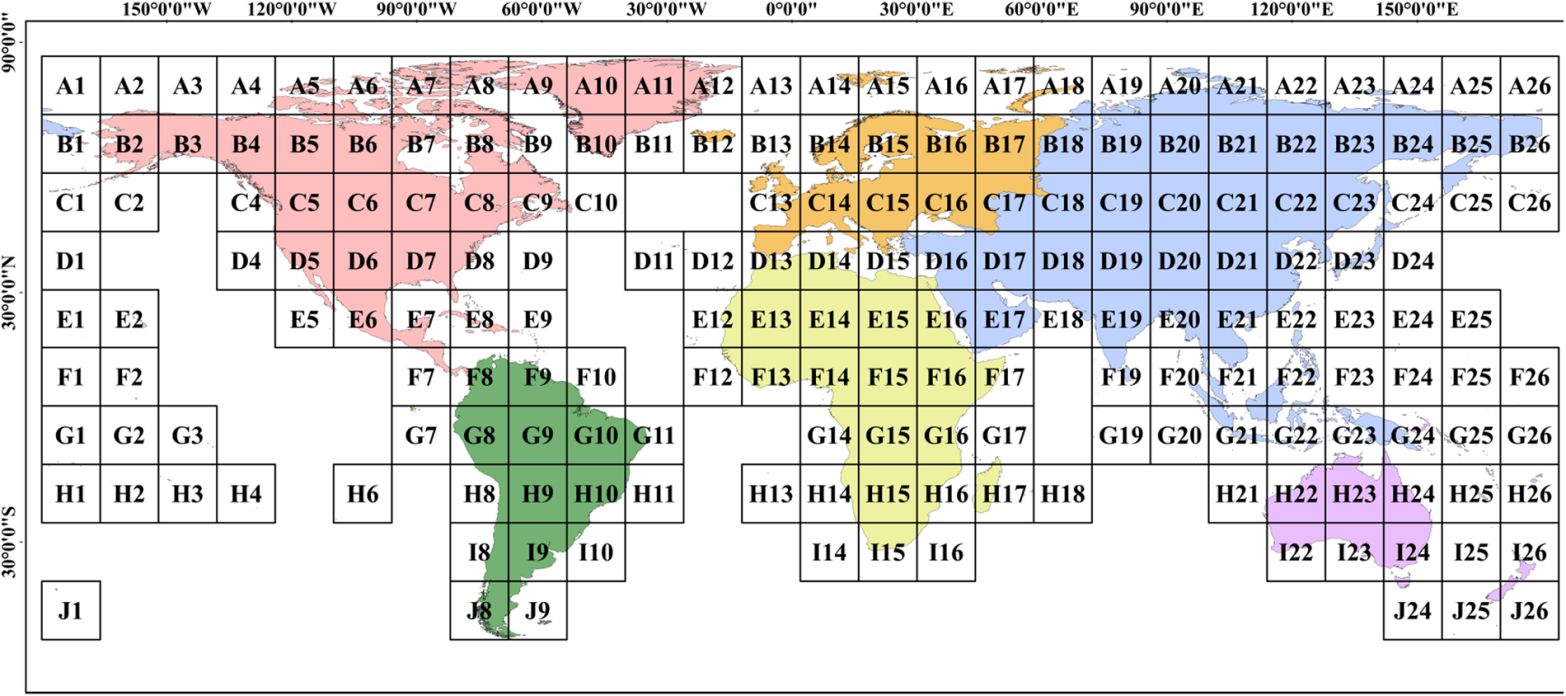
Global elevation data segmentation management (random colors for illustrative 239 purpose).

To address the high computational demand for calculating the LS-factor on a 1-arcsec resolution globally, we merged the single SRTM-D tiles (1° longitude × 1° latitude) into larger tiles with dimensions of 14° longitude ×14° latitude, with careful consideration of memory and computing efficiency. The global elevation data consist of 195 tiles in total, ranging from –180° to +180° longitude and +83° to –56° latitude. The globe was divided into 10 rows from the equator to the poles, denoted as A, B…J, and 26 columns from 180°W to 180°E, denoted as 1, 2…26. The tiles did not overlap, significantly reducing redundancy and thus improving the processing efficiency. Figure 4 shows the global elevation data using the tile labels reported.

### Buffer strategy

**Fig. 5:**
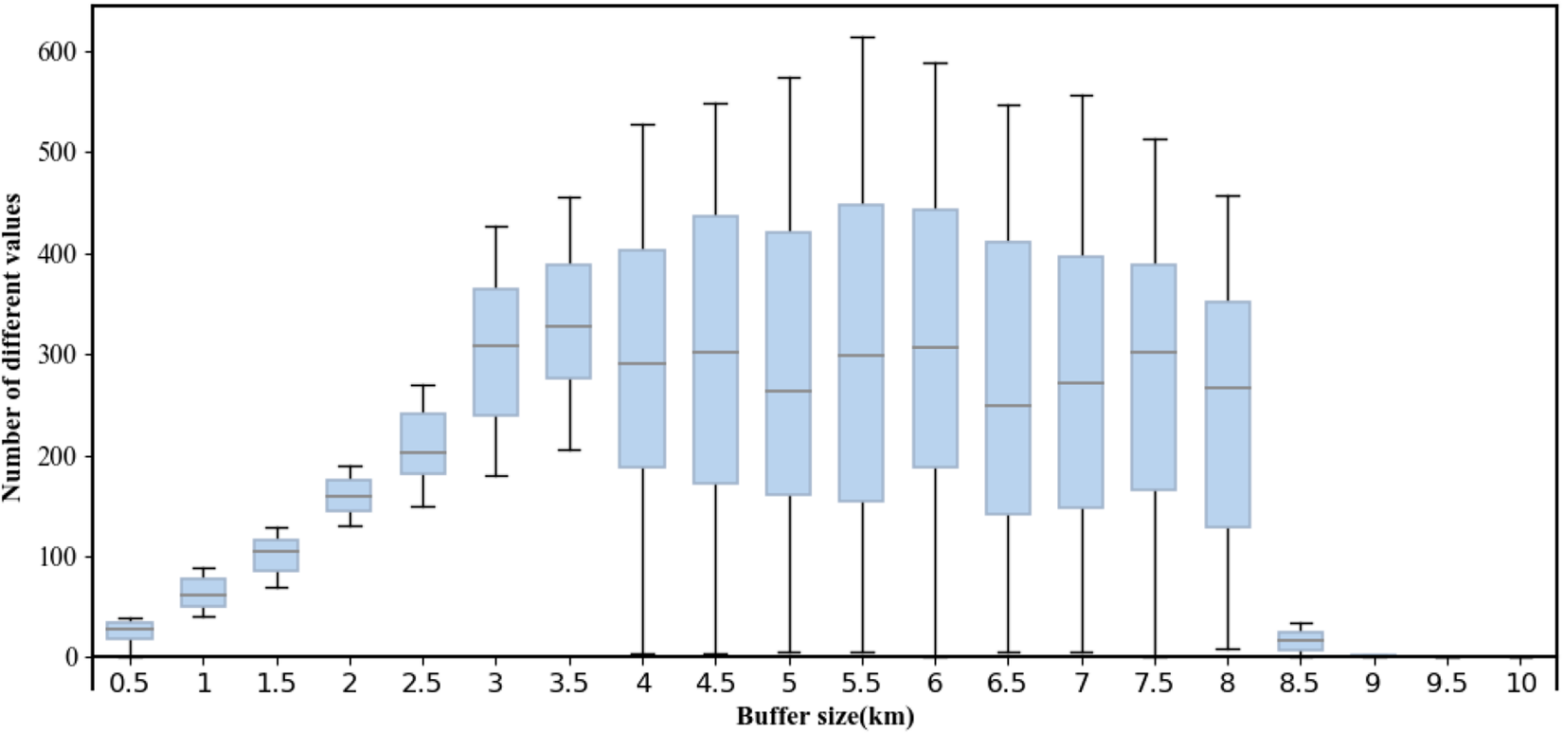
Experiment of Buffer Size. (Distribution of the number of cells in different buffer sizes. Where the bars indicate the number of cells in the quarter to three-quarter range and the horizontal lines in the bars indicate the medians).

A buffer was used to prevent edge information loss by supplementing the tiles divided by the above rules. Considering the influence of the buffer size on large-scale terrain research, a certain buffer size was set for the global-scale LS-factor extraction system, where each tile was extended to a certain distance, and the regular tiles were directly used as unit tiles to extract the slope length one by one. Experiments were conducted to select the buffer and to reduce the error in extracting the slope length. The experiment did not cover all elevation data blocks but instead covered three tiles in a typical area of each continent. These 18 SRTM-D tiles were used to determine a suitable buffer size to represent the global SRTM-D data. The elevation data buffer sizes ranged from 1–10 km, with a step of 0.5 km. The slope length for each block was calculated using various buffer sizes. The same area in the current map was then compared to that in the previous map, and the number of cells was counted for each buffer size (NCBS). If the SRTM-D included the entire basin or subbasin, the buffer size variation in the slope length maps decreased with increasing size. The distribution of the number of cells for the different buffer sizes is shown in Fig. 5. The NCBS increased with buffer size; however, the NCBS began to decrease in some areas when the buffer size reached 3.5 km. Some NCBSs reached zero at a buffer size of approximately 6 km. Finally, all NCBSs decreased to zero at a buffer size of 9 km. According to the buffer size results, we set a 1° (>10 km) buffer size in calculating each block of SRTM-D data, which is sufficient to ensure the global LS calculation accuracy.

### Lat-Lon GCS

**Fig. 6:**
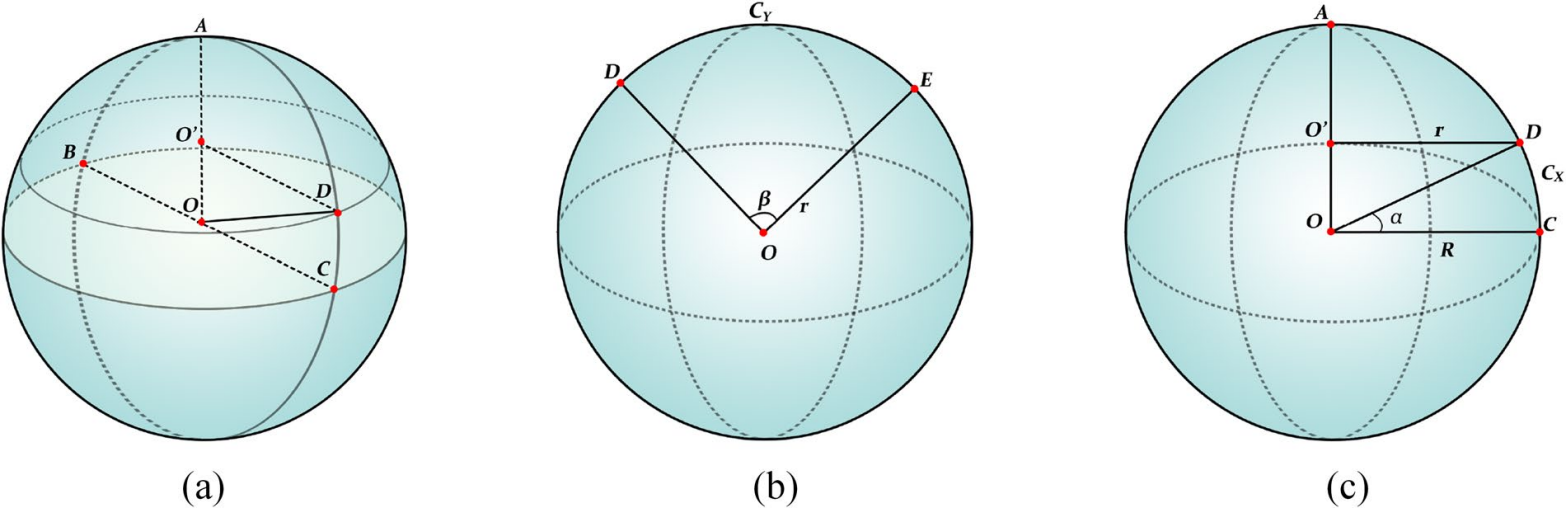
(**a**) Earth sphere model. (**b**),(**c**) Schematic diagram of the longitude and latitude planes of the Earth.

When using raster datasets, the slope steepness and slope length factors can be calculated based on pixels per pixel. This pixelwise analysis approach allows for detailed characterization of the topography across the entire raster dataset. Therefore, the GCS, determined by the horizontal resolution of the DEM, plays a crucial role in determining the accuracy of the slope steepness and slope length calculations. It serves as a fundamental data parameter that influences the precision of extracting these topographic features. Applying ellipsoidal (regular) models of the Earth, the Earth’s surface can be partitioned into a geographically regular grid^38^. Each portion of the Earth’s surface can be represented by cells with the same angular dimensions along the NS and EW directions. Therefore, the size of any grid cell can be calculated from the longitude, latitude, and radius. For example, suppose that the Earth is a perfect sphere^37^, where O denotes the centre of the Earth, and AO denotes the radius of the Earth, as shown in Fig. 6a. The vertical tangent plane, with BC as the axis, represents a meridian plane, as shown in Fig. 6b: R is the average radius of the Earth and D is a point on the surface of the Earth; α is the angle between the point and the equator, which represents the latitude of the point; DC is the spherical distance corresponding to the included angle; r is the radius of the latitude loop where D is located (the latitude surface where D is located is shown in Fig. 6c); DE is the distance along the latitude loop; and m is the longitude difference corresponding to this distance. Then, Eqs. (1) and (2) can be obtained as follows:

1 $${C}_{X}=2\pi R\cdot \alpha /360$$

2 $${C}_{Y}=2\pi r\cdot \beta /360$$

where *C*_*X*_ is the actual distance (m) of $\mathop{DC}\limits^{\frown {}}$ and *C*_*Y*_ is the actual distance (m) of $\mathop{DE}\limits^{\frown {}}$ According to ${\rm{r}}={\rm{Rcos\alpha }}$, we can obtain Eq. (3) as follows:

3 $${C}_{Y}=2\pi R\cdot cos\alpha \cdot \beta /360$$

Because of the same span (unit:°) of the latitude and longitude of the SRTM-D cells, adopting the Earth’s radius R = 6371000 m and β = 1(°)/3600, each SRTM-D grid cell size can be calculated by Eqs. (4) and (5).

4 $${C}_{X}=30.8874791$$

5 $${C}_{Y}={C}_{X}\cdot {\rm{cos\alpha }}$$

where *C*_*X*_ is the GCS along the north‒south direction, which is a constant, at 30.887491 m, and *C*_*Y*_ is the GCS along the east‒west direction, which varies with latitude. Thus, the slope steepness and slope length values in the geographic coordinate system can be derived by combining the parameters of *C*_*X*_, the latitude value of each cell in the SRTM-D data and Eq. 5 with the slope steepness and slope length calculation algorithms.

### Determination of the flow direction, slope steepness, and cell slope length

**Fig. 7:**
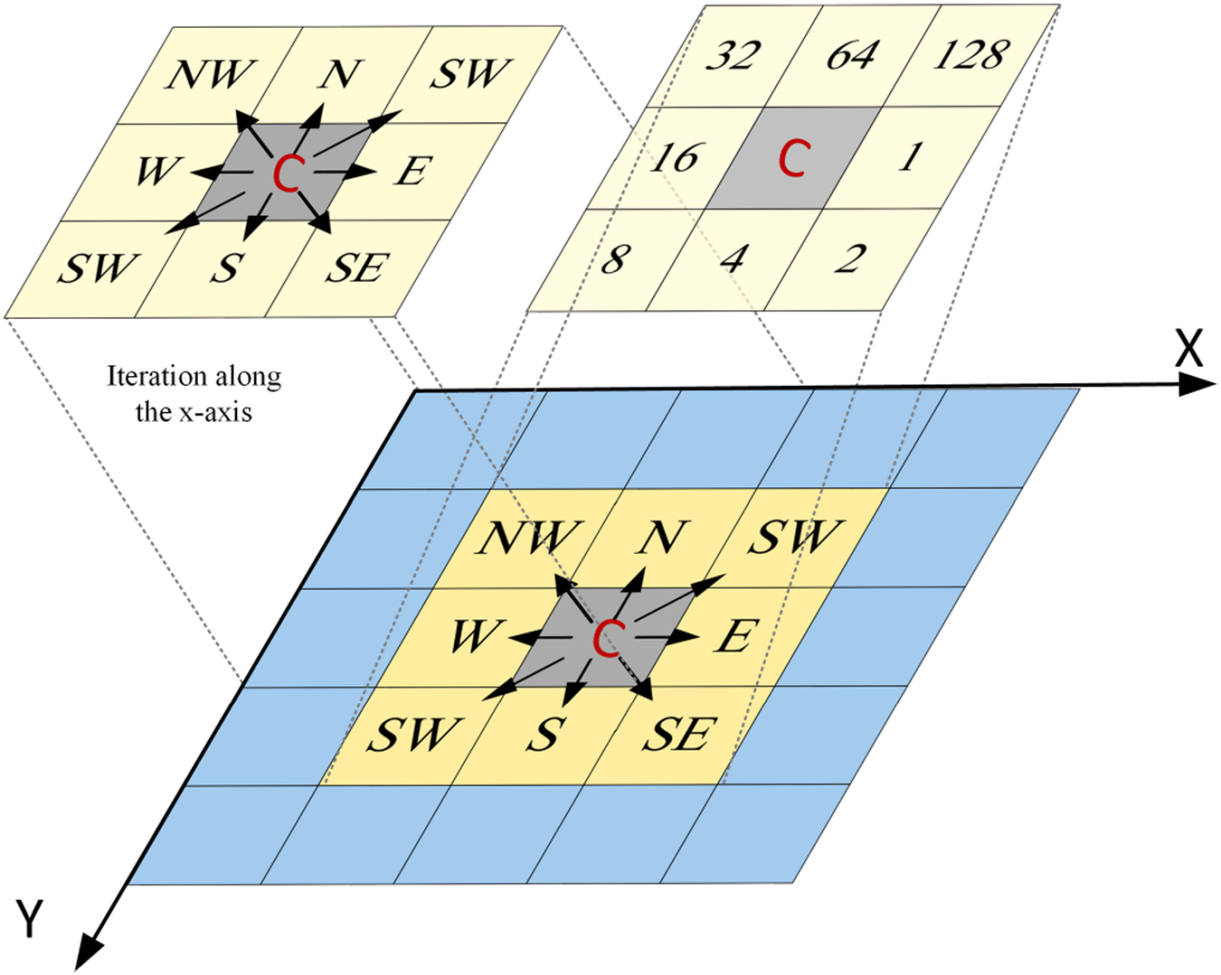
Flow direction of grid and its coding method.

In the analysis of raster datasets for obtaining terrain characteristics, the computation of the flow direction and slope steepness depends on the size of the grid cells and the orientation of the grid. The GCS, determined by the spatial resolution, influences the precision of these calculations, with smaller cells offering higher-accuracy topographic details. Additionally, the grid orientation, often specified by the coordinate system, plays a role in accurate flow and slope assessments, particularly in regions with diverse topography. The slope steepness and flow direction were calculated using the D8 algorithm^50,51^ based on the steepest slope descent concept. The flow distribution principle of the D8 algorithm suggests that on a 3 × 3 DEM grid, the outflow direction refers to the direction of the neighbouring cell with the maximum downward slope steepness. The maximum downhill slope steepness among the eight surrounding directions was adopted as the cell slope steepness; moreover, as previously mentioned, the direction of this cell was adopted as the outflow direction^52^. As shown in Fig. 7, C is the location of the current cell, and its outflow direction is that of one of the eight surrounding cells, marked as 1, 2, 4, 8, 16, 32, 64, and 128.

The basic principle of the grid slope steepness calculation, using the D8 algorithm, is to adopt the central grid cell as the grid to be calculated and determine the difference in the distance-weighted elevation between the central grid and its eight directions. The grid slope steepness can be calculated by Eq. 6. In addition, to ensure that each cell is connected to the river network, the slope steepness of the grid cell was set to 0.1 at a slope steepness of 0.

6 $$S=Max\left(\arctan \left(\frac{{Z}_{c}-{Z}_{i}}{g}\right)\right)$$

where S denotes the slope steepness of the central grid to be calculated, *Z*_*c*_ denotes the elevation value of the central grid, *Z*_*i*_ denotes the grid elevation value in the neighbourhood of the central grid, and g denotes the distance between the two grid cells to be calculated. The value of g is related to the positional relationship between the central grid and the adjacent grid, which can be divided into three cases: when one grid is located at the south or north (S or N, respectively) position of another grid, g = *C*_*X*_; when one grid is located at the east or west (E or W, respectively) position of another grid, g = *C*_*Y*_; and when it is located at the southeast, southwest, northwest, or northeast (SE, SW, NW, or NE, respectively) position of another grid,

$${\rm{g}}=\sqrt{{C}_{X}^{2}+{C}_{Y}^{2}}.$$

The cell slope length (CSL) is the distance from the centre grid to the next grid along the flow direction, which depends on the size of the cells and the travel direction between the cells. In the case of D8 algorithm application, the CSL can be calculated in the same manner as g.

### Calculation of the cumulative slope length

The slope length is defined as the horizontal distance from the starting point along the vertical contour line to the slope deposit or obvious channel^53^. When calculating based on grid data, the slope length can be calculated by accumulating the CSL along the slope steepness direction until the endpoint of the slope length cut-off is reached. This accumulation process may involve thousands of grid cells. As the calculation process generates a cumulative effect, it is denoted as the cumulative slope length. The cumulative slope length can be calculated by Eq. (7):

7 $${\lambda }_{i,j}=\mathop{\sum }\limits_{x=0,y=0}^{x=i,y=j}\,\mathop{\sum }\limits_{k=1}^{m}{\lambda }_{c}$$

where *λ*_*i,j*_ denotes the slope length of the grid cell with coordinates (i, j), *λ*_*c*_ denotes the CSL of each grid, m is the slope length exponent, and k denotes the eight surrounding cells with coordinates (i, j).

In this study, the end of the slope length was determined by two factors that define the slope length: the slope cut-off point and the channel network. The relationship between the slope steepness change rate and the cut-off factor determines the slope cut-off^54,55^. For example, considering a slope steepness of 5% (approximately 2.861°) as the dividing point, when the value is less than 5%, the cut-off factor is set to 0.7; when it is greater than or equal to 5%, the cut-off factor is set to 0.5^12^. When the slope steepness change rate was higher than the cut-off factor, the point was marked as a cut-off point. The cut-off point of the channel network was determined by setting the threshold for the catchment area. When the catchment area was greater than the threshold, the point was marked as a cut-off point.

The calculation of the cumulative slope length begins with the starting grid cell, accumulating the value along the maximum slope steepness direction among the surrounding 8 directions. However, for the SRTM-D data, the maximum slope length along a certain flow path cannot be determined. Therefore, it is necessary to calculate the cumulative slope length from the grid cell starting point in a point-by-point manner and perform the forward-reverse traversal operation^12^.

### Calculation of the LS-factor

**Fig. 8:**
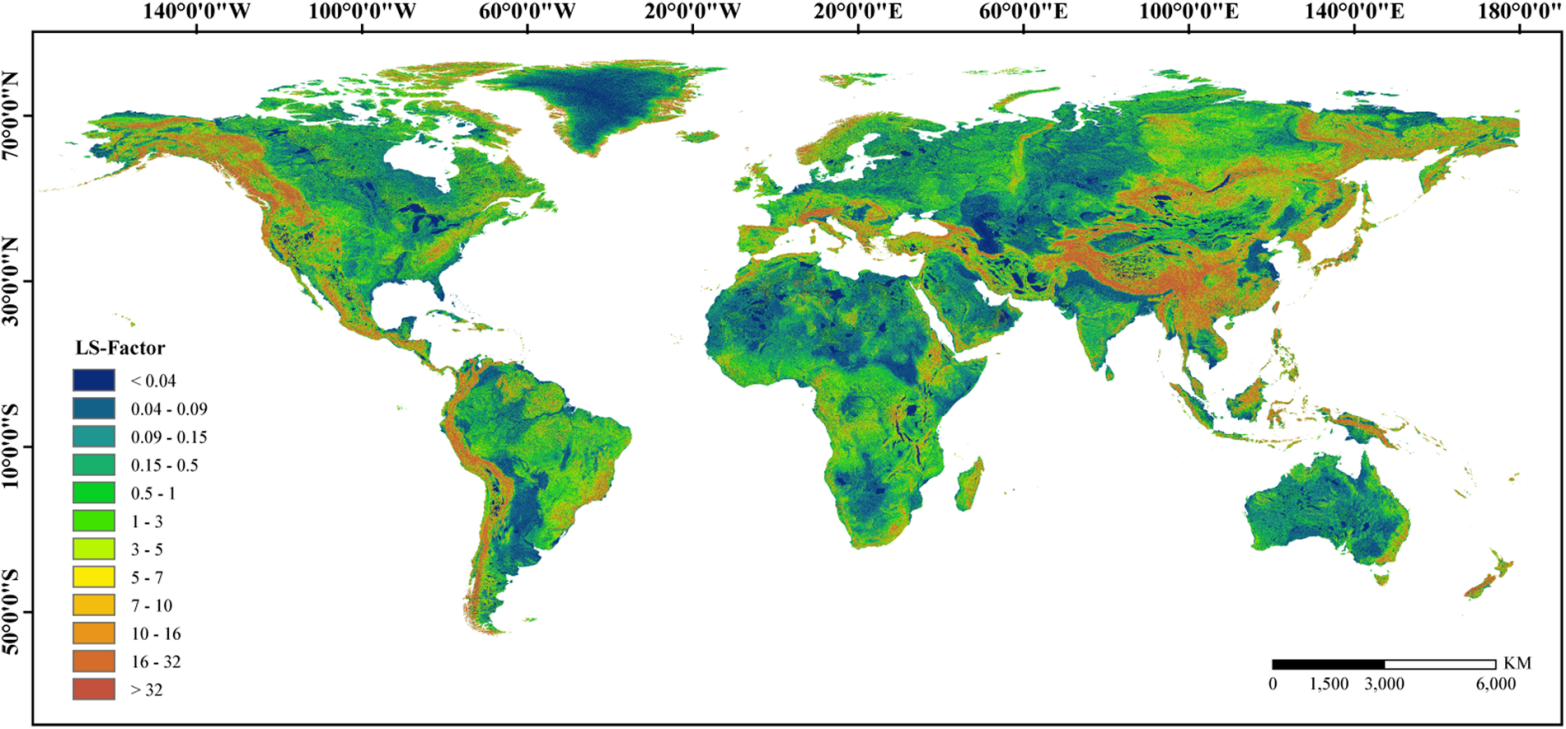
Spatial pattern of extraction result of global LS-factor.

The USLE/RUSLE is the most frequently used equation for soil erosion estimation, and the CSLE was extended from the USLE and RUSLE, which is a more suitable soil erosion equation for soil environments with steep slopes (>10°). The difference between the USLE/RUSLE and CSLE is that the former divides the slope into two grades, while the latter divides it into three grades. It has been demonstrated that the S-factor calculated using the USLE/RUSLE could be lower by approximately 20% on a regional scale^56^. McCool *et al*.^57^ found that soil loss occurred faster on steeper slopes. Considering that many places worldwide exhibit a slope steepness higher than 10°, the CSLE was used to calculate the global LS-factor so that the slope steepness could be determined more accurately. In the CSLE, the slope length and steepness jointly determine the erosion topographic factor^58^. To avoid the error caused by considering only a uniform slope length, the segmented slope length factor equation was used to calculate the slope length factor. The LS-factor can be calculated by Eqs. (8–10). A global representation of the LS-factor layer produced using this methodology is shown in Fig. 8.

8 $$S=\left\{\begin{array}{lc}10.8\,{\sin }\,\theta +0.03 & \theta  < {5}^{\circ }\\ 16.8\,{\sin }\,\theta -0.05 & {5}^{\circ }\le \theta  < 1{0}^{\circ }\\ 21.91\,{\sin }\,\theta -0.96 & \theta \ge 1{0}^{\circ }\end{array}\right.$$

9 $$L=\left\{\begin{array}{cc}\frac{{\lambda }_{out}^{m+1}-{\lambda }_{in}^{m+1}}{\left({\lambda }_{out}-{\lambda }_{in}\right){(22.13)}^{m}} & {\lambda }_{out}-{\lambda }_{in} > 0\\ {\left(\frac{{\lambda }_{out}}{22.13}\right)}^{m} & {\lambda }_{out}-{\lambda }_{in}\le 0\end{array}\right.$$

10 $${\rm{in}}\,{\rm{which}}\,{m}=\left\{\begin{array}{l}0.2\,\theta \le 0.{5}^{\circ }\\ 0.3\,0.5\le \theta  < 1.{5}^{\circ }\\ 0.4\,1.5\le \theta  < {3}^{\circ }\\ 0.5\,{3}^{\circ }\le \theta \end{array}\right.$$

where θ is the slope steepness (°), S is the slope steepness factor, λ_in_ denotes the slope length at the inlet, λ_out_ denotes the slope length at the outlet, m is a variable length-slope exponent, and L is the slope length factor.

## Validation Methods

Three approaches were used to validate the performance of the LS-WPC method: (1) the Himmelblau–Orlandini mathematical surface (HOMS), (2) SRTM-D data containing five landform types (flat, basin, hill, mountain and plateau areas), and (3) a previously published continent-scale LS-factor dataset, including Australia and the European Union.

### HOMS

**Fig. 9:**
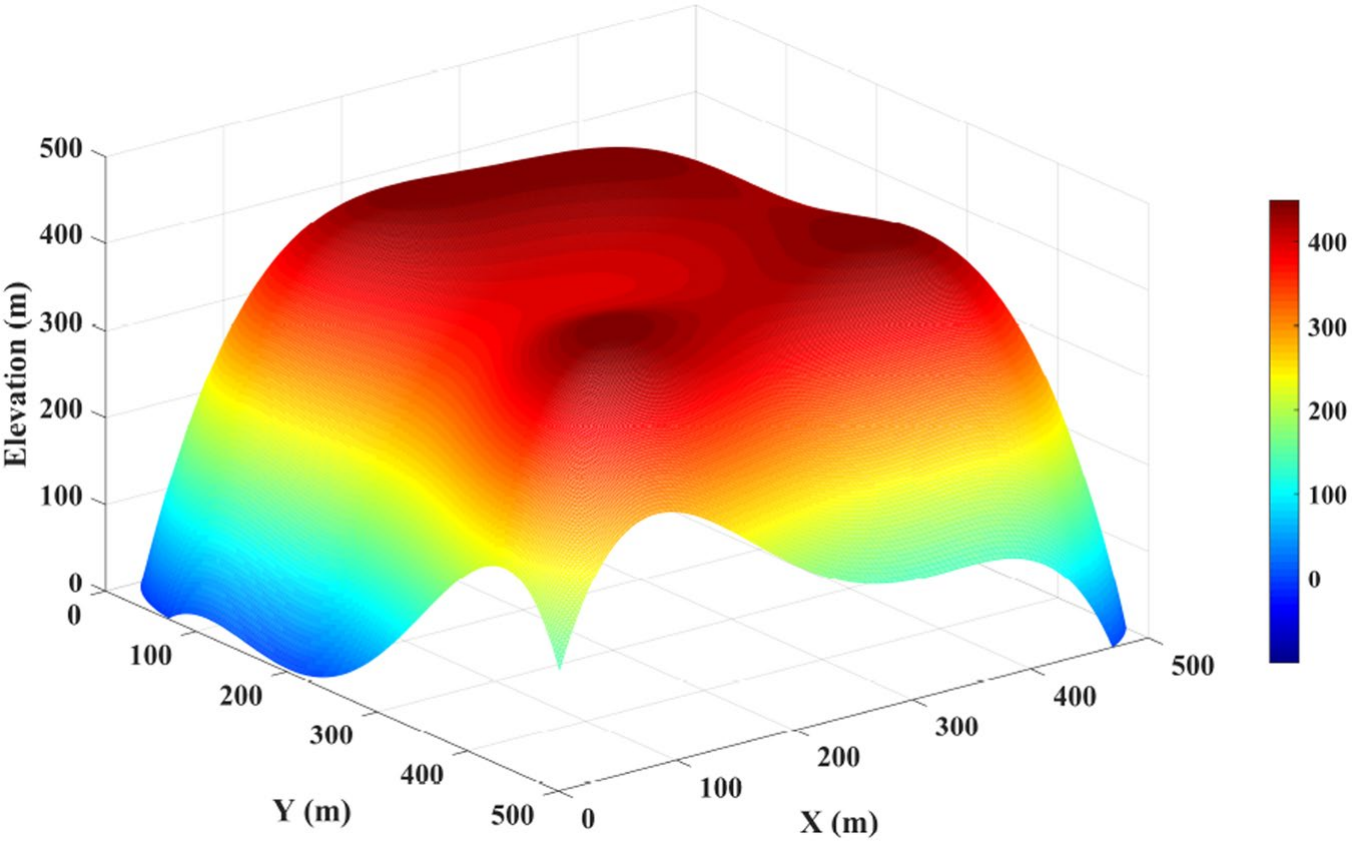
Himmelblau-Orlandini surface with four local hilltops, three saddle points, and a flow convergence area. (Note: the above continuous surface was discretized into raster data at an interval of 0.1 and then enlarged tenfold as a whole. The resolution of the synthetic surfaces was thus 1 m).

In evaluation, it is crucial to adopt an objective and data-independent methodology^44^. Utilizing DEMs defined by mathematical surfaces can effectively eliminate data errors, thereby ensuring that the observed errors are solely attributable to algorithmic factors^59^. Therefore, we employed the HOMS model^60^ to validate the performance of the LS-WPC method. The HOMS is a discrete surface generated using the Himmelblau function and after-affine transformation, which has concave and convex surfaces, a divergent collection, and other mathematical features. The HOMS can simulate a relatively complex surface, with four local hilltops, three saddles, and a flow convergence area (Fig. 9). The HOMs can be expressed as Eq. (11):

11 $${Z}_{\left(x,y\right)}=45-0.075\left[{\left\{{\left(\frac{x-25}{5}\right)}^{2}+\left(\frac{y-25}{5}\right)-4\right\}}^{2}+{\left\{{\left(\frac{y-25}{5}\right)}^{2}+\left(\frac{x-25}{5}\right)-7\right\}}^{2}\right]$$

where x ∈ [0,50], y ∈ [0,50], and Z is the elevation of (x, y). Notably, x, y, and z are in units of metres.

### SRTM-D data containing five landform types

**Fig. 10:**
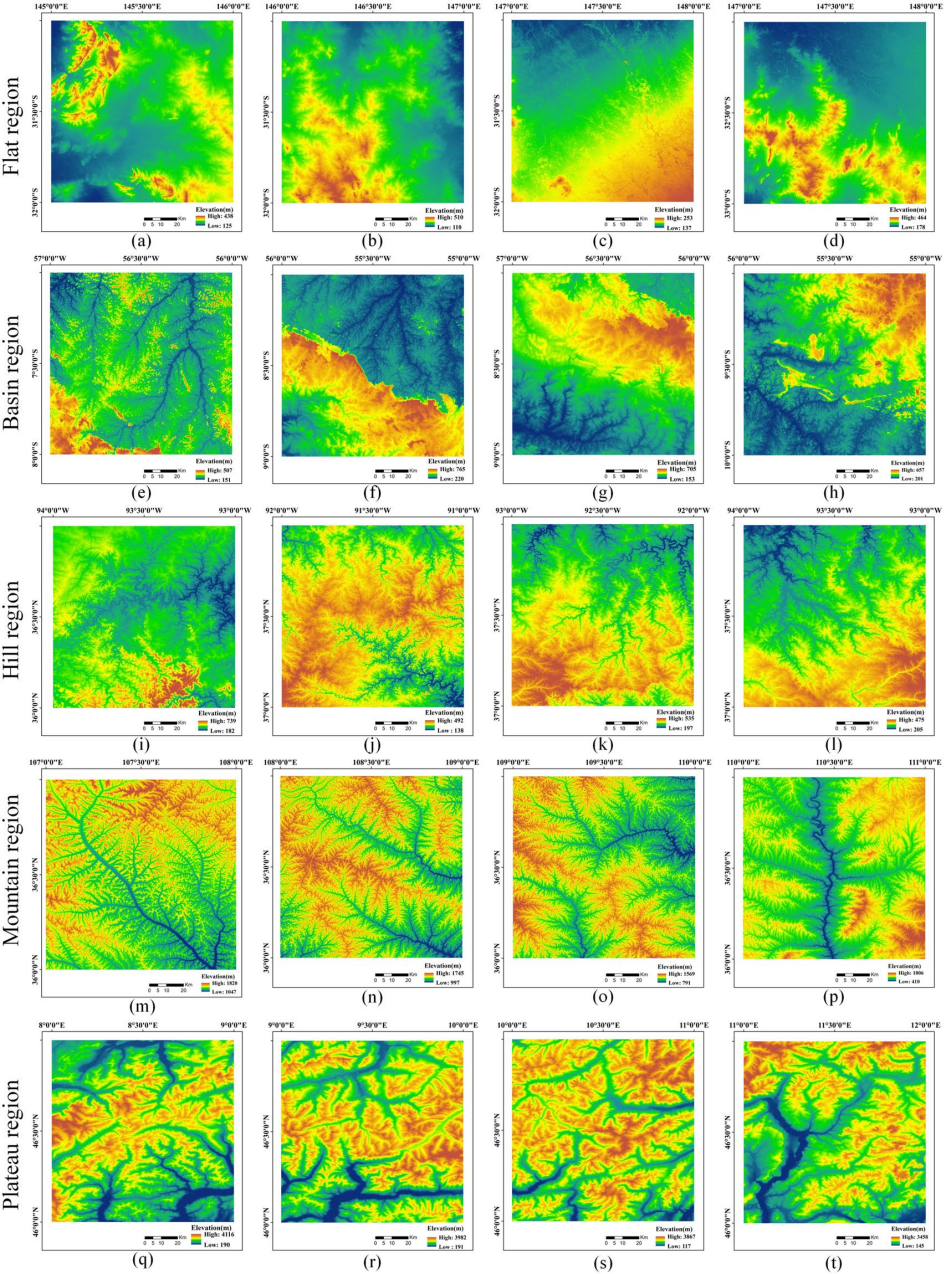
SRTM-D datas with five landform types, including flat, basin, hill, mountain, and plateau regions.

The validation of a global DEM must rely on many test cases with different landscapes or on simulations to meet multiple requirements^61^. A given landform type is distinguished by its dimensions and by the statistical frequency of its principal geomorphic attributes. These include the slope length, gradient and frequency distribution, the frequency of slope inflections or reversals, and the magnitude of the internal relief^62^.Thus, the criteria for selecting SRTM-D data were based on two main factors: (1) the availability of high-precision reference models (5 m) and (2) the representation of diverse topographic conditions, including flats, hills, basins, mountains, and plateau areas. The size of each sample was 1° × 1°, and in total, more than 259 million pixels were analysed. The elevation data of the samples are shown in Fig. 10.

### Previously published LS-factor datasets

Two previously published LS-factor datasets were compared with the DS-LS-GS1 dataset. One dataset is the seamless LS-factor digital map for Australia (DS-LS-AU) with a spatial resolution of 1 arcsecond based on the SRTM-D data^63^. The other is the LS-factor dataset for the European Union (DS-LS-EU) based on the 1-arcsec DEM, a hybrid product based mainly on the SRTM-D and ASTER GDEM^56^. Both LS-factor datasets showed significant improvements in past assessments owing to the higher input data accuracy.

## Data Records

**Fig. 11:**
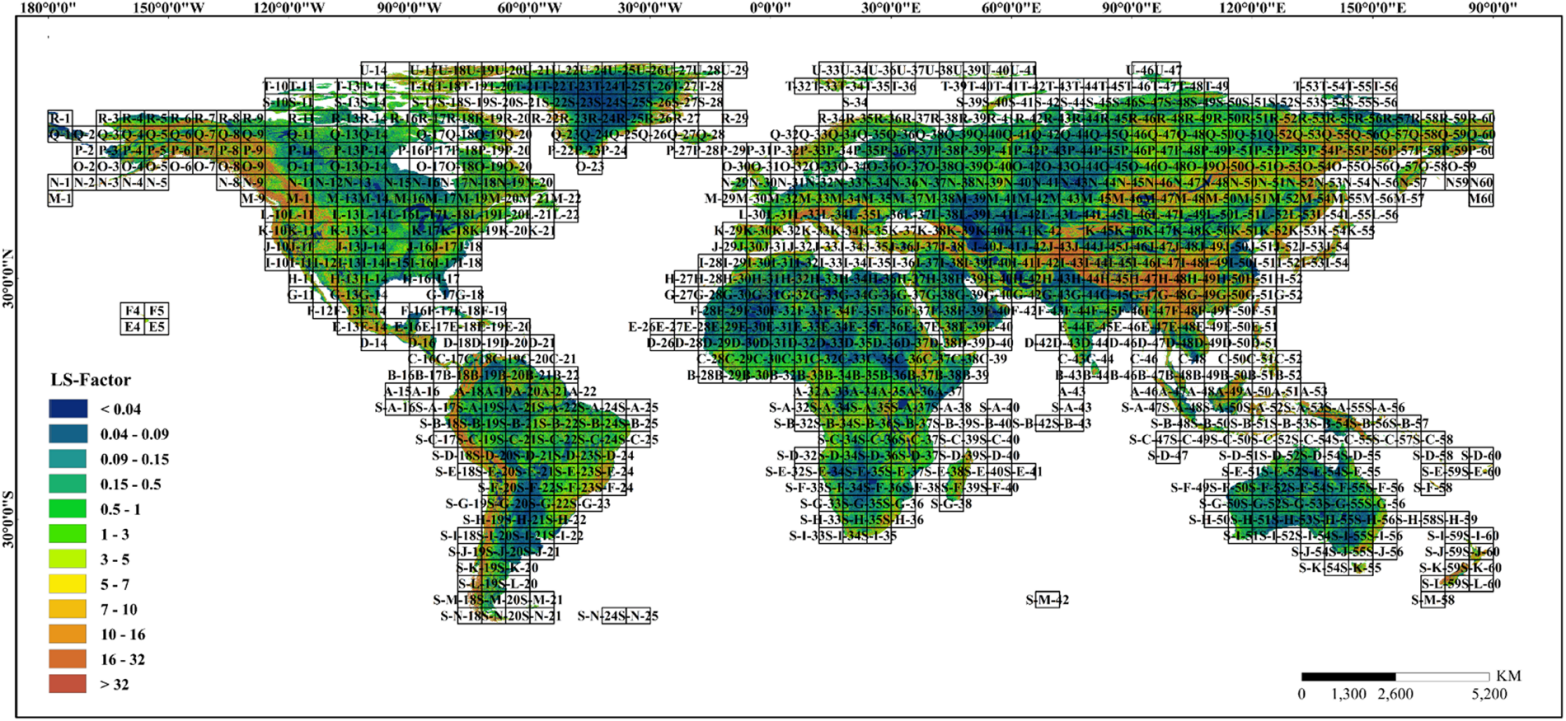
1-arcsec global LS-factor dataset segmentation management.

The global-scale and 1-arcsec resolution LS-factor dataset^64^ is available at 10.11888/Terre.tpdc.300613 (please refer to the Supplementary File 1-Data link usage instructions). We split the entire LS-factor dataset into 1060 tiles of the same size. The rules for dividing the data were based on standard division of a 1:1 million measuring scale. A representation of the global LS-factor dataset with a 1-arcsec resolution using tile labels is shown in Fig. 11. The dataset was named according to latitude-longitude and stored in GeoTIFF format. To reduce the file size, the data were compressed and stored in zip format. They can be downloaded, uncompressed, and then viewed using various GIS software programs.

## Technical Validation

First, we generated a HOMS based on the SRTM-D data in the 0-latitude region (SRTM-Dlat00) as the GCS here is the closest along the north‒south and east‒west directions, and the sample data are the least affected by the coordinate difference. In addition, SRTM-Dlat30, SRTM-Dlat40, and SRTM-Dlat50 denote the HOMSs located in the 30, 40, and 50 latitude zones, respectively, which were used to study the influence of the LS-WPC method on the LS-factor extraction results at different latitudes.

The three topographic attributes (slope steepness, slope length, and LS-factor) extracted by the LS-WPC method and the LS-factor extraction algorithm in the projected coordinate system (LS-PCS) were compared from the aspects of the spatial pattern (geographical distribution) and basic feature statistics. The standard deviation (SD) and absolute deviation (AD) were used to determine the calculation error. These metrics can be obtained by Eqs. (12) and (13), respectively:

12 $$SD=\sqrt{\frac{1}{N}\mathop{\sum }\limits_{1}^{N}{\left(L{S}_{a}-L{S}_{b}\right)}^{2}}$$

13 $$AD=\frac{1}{N}\mathop{\sum }\limits_{1}^{N}\left|{{\rm{LS}}}_{{\rm{a}}}-{{\rm{LS}}}_{{\rm{b}}}\right|$$

where N is the number of grid cells, LS_a_ is the LS-WPC calculation result, and LS_b_ is the LS-PCS calculation result.

In addition, in terms of SRTM-D data, the local 5-m high-resolution reference models were resampled to 1-arcsec, and the slope steepness, slope length and LS-factor values calculated on the basis of these data were adopted as the true values. The calculation results of the LS-WPC and LS-PCS methods were compared with the measured results, and the SD, AD, and correlation coefficient (R^2^) were used to evaluate the errors.

Finally, we used the coefficient of variation (CV) to evaluate the performance of our LS-factor dataset by comparing it with previously published data. The CV is an indicator of the degree of heterogeneity within the data and is calculated from the ratio of the SD to the average value.

### Evaluation of the HOMS extraction results

**Fig. 12:**
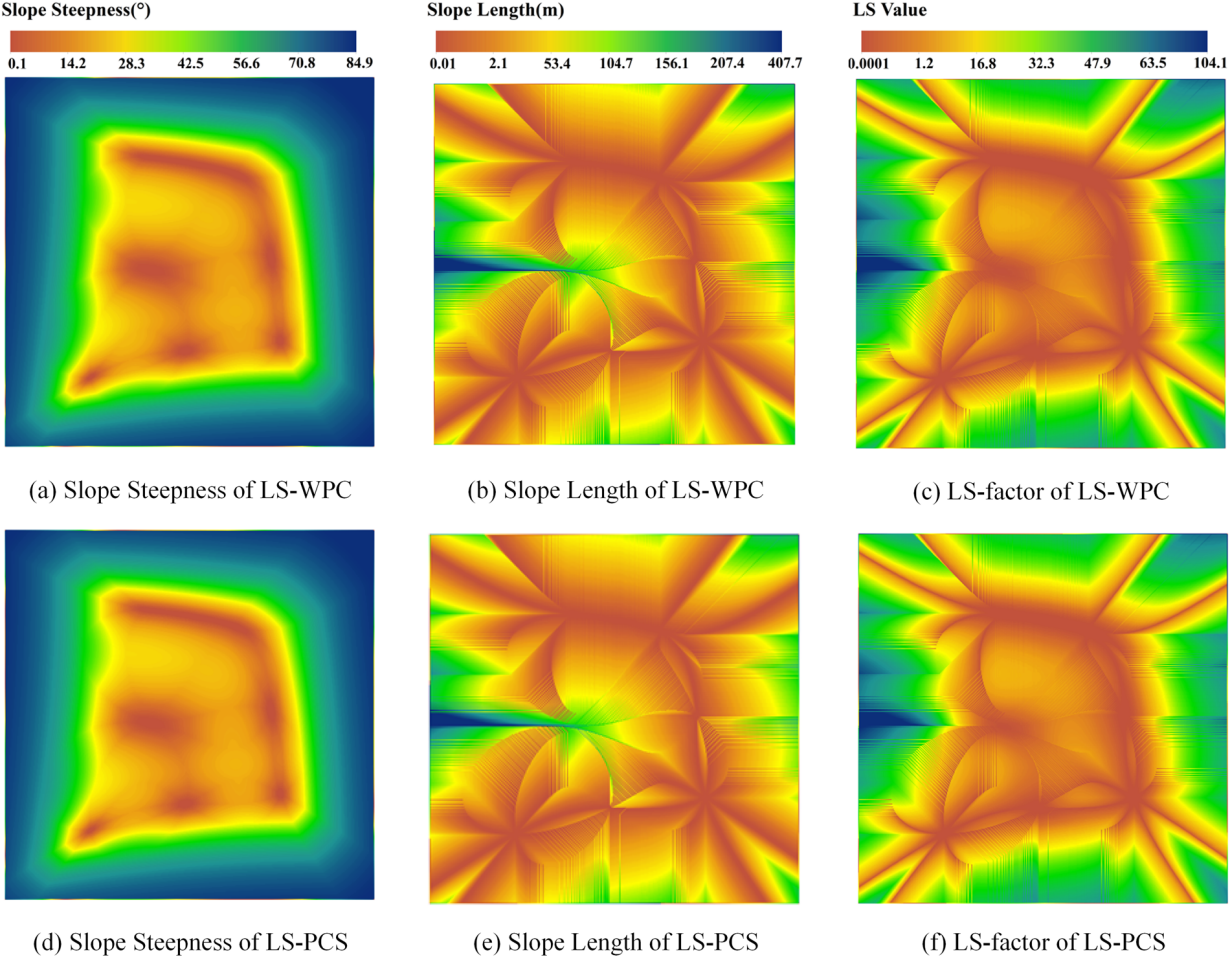
Topographic factors extraction results in HOMS.

The LS-WPC and LS-PCS calculation results are shown in Fig. 12. The results of the LS-WPC method showed that the maximum slope steepness was 84.97° and that the minimum was 0.1°, with the average slope steepness reaching 50.91°. The LS-PCS method results showed that the maximum, minimum, and mean slope steepness values were 84.97°, 0.1°, and 50.89°, respectively (Table 1). High slope steepness values were distributed in the steep-slope area outside the four local high points, while the change in the slope inside the local high points was not obvious (Fig. 12a,d). Considering only the slope cut-off case, the maximum, minimum, and mean slope lengths of the LS-WPC method were 407.65, 0.48, and 64.96 m, respectively; the maximum, minimum, and mean slope lengths of the LS-PCS method were 407.68, 0.48, and 64.98 m, respectively (Table 1). The slope length is accumulated from the local high point along the direction of the steepest slope change and can be accumulated at the watershed boundary of the converging slope, which can reflect the surface relief (Fig. 12b,e). The maximum, minimum, and mean values of the LS-factor of the LS-WPC method were 133.12, 0.01, and 36.90, respectively, while those of the LS-PCS method were 133.62, 0.01, and 37.2, respectively (Table 1). The LS-factor is affected by both the slope length and slope steepness and is consistent with the slope steepness distribution overall (Fig. 12c,f). The texture characteristics of the two methods were highly consistent, and the mean and SD values of the three topographic indices were highly similar.

**Fig. 13:**
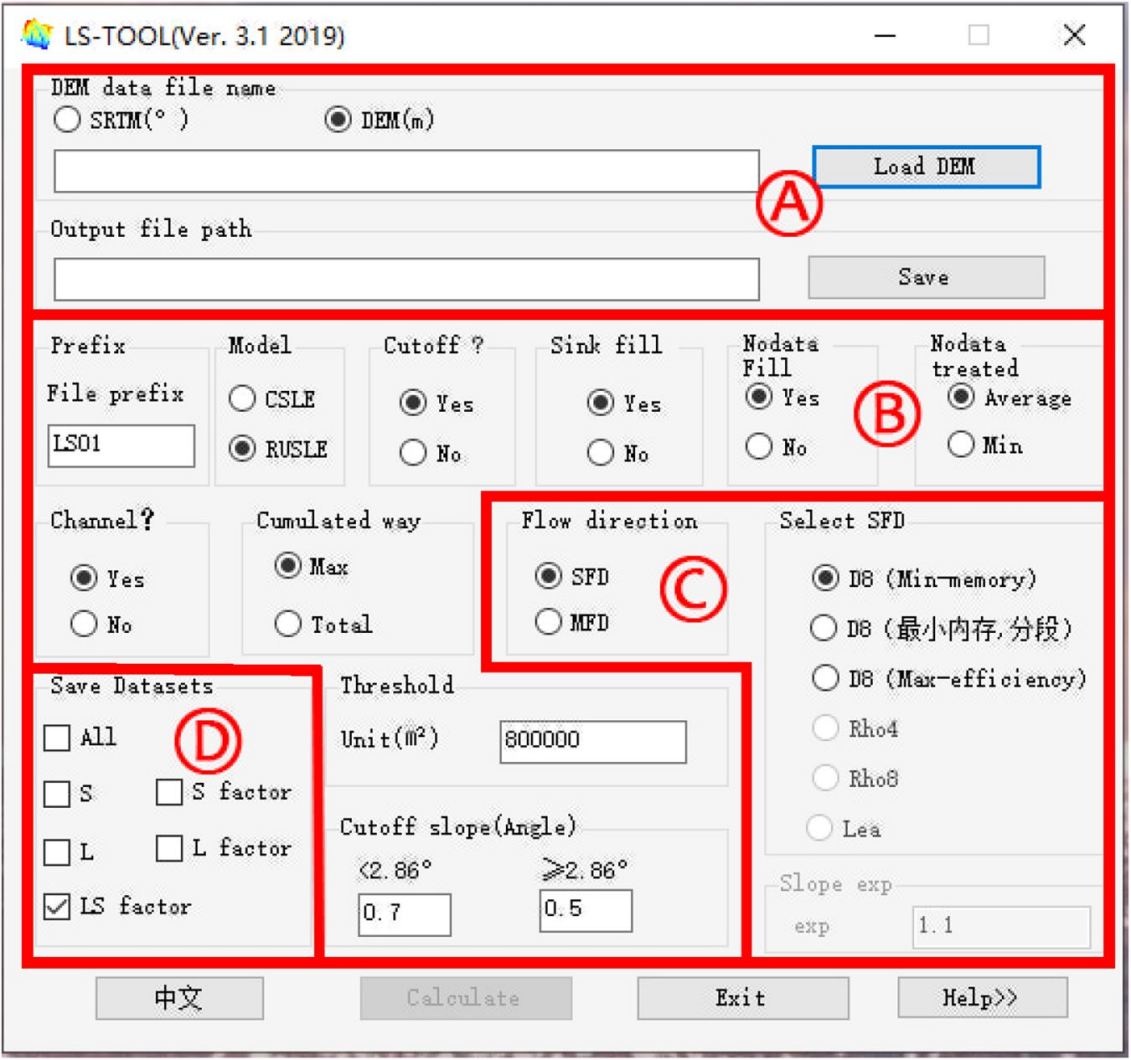
The graphical user interface (GUI) of LS-TOOL.

**Table 1:** Comparison of topographic factors results between LS-WPC and LS-PCS in SRTM-Dlat00.

Comparative item		Min	Max	Mean	SD
slope steepness (${}^{\circ }$)	LS-WPC	0.10	84.97	50.91	25.09
	LS-PCS	0.10	84.97	50.89	25.09
slope length (m)	LS-WPC	0.48	407.65	64.96	49.01
	LS-PCS	0.48	407.68	64.98	49.03
LS-factor	LS-WPC	0.01	133.12	36.90	24.82
	LS-PCS	0.01	133.63	37.20	24.83

To demonstrate the impact of each of these two algorithms on the calculation of topographic factors, both the SD and AD were calculated, and the results are listed in Table 2. The SD and AD of the slope steepness were 0.001 and 0.124, respectively; the SD and AD of the slope length were 0.138 and 0.166, respectively; and the SD and AD of the LS-factor were 0.701 and 0.704, respectively. In summary, there were small differences among the three topographic indices.

**Table 2:** Difference of topographic factors results between LS-WPC and LS-PCS in SRTM-Dlat00.

Comparative item	SD	AD
Slope Steepness (${}^{\circ }$)	0.001	0.124
Slope Length (m)	0.138	0.166
LS-factor	0.701	0.704

The calculated results for the HOMS at the different latitudes were statistically analysed. Table 3 shows that with increasing latitude, the average slope steepness exhibited an increasing trend, whereas the average slope length and LS-factor exhibited a decreasing trend. The mean LS-factor value is 34.85 at latitude 50, which is 2.05 lower than the value of 36.90 at latitude 0. With increasing latitude, the cell size decreased along the transmeridional direction, which caused an increase in the slope steepness along the transmeridional direction, resulting in an overall increase in the slope steepness and a decrease in the transmeridional slope length, further resulting in an overall decrease in the slope length. There were differences in the LS-factor extraction results at the different latitudes; however, the overall results were similar because the flow direction matrix did not change, and the slope cut-off was consistent.

**Table 3:** Comparison of LS-WPC calculation results in different latitudes.

Comparative item		Min	Max	Mean	SD
Slope Steepness (${}^{\circ }$)	SRTM-Dlat00	0.10	84.97	50.91	25.09
	SRTM-Dlat30	0.10	85.30	52.11	25.11
	SRTM-Dlat40	0.10	85.52	53.34	25.09
	SRTM-Dlat50	0.10	85.77	54.99	24.99
Slope Length (m)	SRTM-Dlat00	0.48	407.65	64.96	49.01
	SRTM-Dlat30	0.42	365.41	61.12	46.07
	SRTM-Dlat40	0.37	354.79	57.84	43.78
	SRTM-Dlat50	0.31	354.79	53.57	41.35
LS-factor	SRTM-Dlat00	0.01	133.12	36.90	24.82
	SRTM-Dlat30	0.01	126.42	36.25	23.93
	SRTM-Dlat40	0.01	124.61	35.68	23.10
	SRTM-Dlat50	0.01	124.61	34.85	22.04

### Evaluation of the extraction results for the SRTM-D data

The statistical results of the slope steepness, slope length, and LS-factor in the real terrain areas are listed in Supplementary Tables 3–5. The highest average values of the slope steepness were observed in the plateau regions, followed by the mountain, hilly, basin, and flat regions. The distributions of the slope length and LS-factor were consistent with that of the slope steepness. The calculated results were consistent with the terrain characteristics and the results from the literature^12^.

The difference in the mean LS-factor between the two methods was less than 0.4 (Supplementary Table 5). According to the five landform types, the comparison results between the two methods and the true values in the real terrain sample areas are listed in Supplementary Table 6. The correlation between the results of the two methods and the true values was close. The correlation for the slope steepness was better than that for the slope length and LS-factor. A possible reason is that the error in calculating the slope steepness was not accumulated; it only occurred for one grid, while for the slope length, the error was accumulated from the starting point along the flow path until the end of the grid. Moreover, the R^2^ value of the LS-WPC method was overall higher than that of the LS-PCS method, which indicates that the LS-WPC method results better agree with the actual values. From the perspective of the calculation error, the SD and AD values of the LS-PCS method were higher than those of the LS-WPC. The main reason is that projection conversion led to elevation changes and grid point offsets, which could cause a chain reaction in the subsequent calculation.

### Comparison with the DS-LS-AU and DS-LS-EU datasets

A comparison of the CV between the DS-LS-AU and DS-LS-GS1 datasets is shown in Table 4, and a comparison of the CV between the DS-LS-EU and DS-LS-GS1 datasets is shown in Table 5. The CVs of these LS-factor datasets are highly consistent. The CV of the DS-LS-GS1 dataset is slightly higher than that of DS-LS-EU and DS-LS-AU datasets overall, and the error remains within the allowable range. This may be due to the errors caused by projection conversion and the choice of different soil erosion models. In addition, we obtained the CV for the remaining 205 countries on six continents (Supplementary Tables 7–12). The most significant variation was noted in France, Hungary, and Poland, whereas the lowest variation was noted in the Baltic States, Luxembourg, and the Netherlands. The aggregated data allowed for quick estimation of the influence of the LS-factor on the overall soil loss rate in a country^56^. These parameters could help researchers quickly select important global hotspots for watershed management, shoreline protection, and riverbank protection.

**Table 4:** Comparison of CV between DS-LS-AU and DS-LS-GS1.

Country	Datasets	CV
Australia	DS-LS-AU	3.49
	DS-LS-GS1	3.60

**Table 5:** Comparison of CV between DS-LS-EU and DS-LS-GS1.

Country	Datasets	CV
Austria	DS-LS-EU	1.14
	DS-LS-GS1	1.35
Bulgaria	DS-LS-EU	1.28
	DS-LS-GS1	1.64
Croatia	DS-LS-EU	1.36
	DS-LS-GS1	1.95
Cyprus	DS-LS-EU	1.18
	DS-LS-GS1	1.53
France	DS-LS-EU	1.81
	DS-LS-GS1	2.40
Germany	DS-LS-EU	1.57
	DS-LS-GS1	2.42
Greece	DS-LS-EU	1.07
	DS-LS-GS1	1.34
Italy	DS-LS-EU	1.34
	DS-LS-GS1	1.58
Luxembourg	DS-LS-EU	1.04
	DS-LS-GS1	1.55
Malta	DS-LS-EU	1.46
	DS-LS-GS1	1.42
Poland	DS-LS-EU	1.67
	DS-LS-GS1	3.06
Slovenia	DS-LS-EU	1.11
	DS-LS-GS1	1.44
Greece	DS-LS-EU	1.07
	DS-LS-GS1	1.34

### Efficiency validation

Table 6 provides the running times for both the LS-WPC and LS-PCS methods. Based on the analysis of actual terrain samples, it was observed that the computational time of the LS-PCS method increased with increasing elevation data range. This could be attributed to the linear increase in the projection conversion time with increasing number of grids. In contrast, the LS-WPC method effectively reduced the projection conversion time, leading to an improved computational efficiency.

**Table 6:** Efficiency comparison of LS-WPC and LS-PCS methods.

Sample Range	Method	Raster Number	Running Time(s)			Efficiency Improvement (%)
			Transforming Coordinate	Calculating LS	Total Time	Ratio
1° × 1°	LS-PCS	3499 × 4122	6.24	35.88	42.11	20.47
	LS-WPC	3601 × 3601	0.00	33.50	33.49	
2° × 2°	LS-PCS	7730 × 8839	27.66	189.97	217.64	22.04
	LS-WPC	7201 × 7201	0.00	169.67	169.67	
3° × 3°	LS-PCS	10541 × 12332	61.33	522.91	584.24	22.76
	LS-WPC	10801 × 10801	0.00	451.29	451.29	
5° × 5°	LS-PCS	17665 × 20462	252.43	1638.56	1890.99	25.52
	LS-WPC	18001 × 18001	0.00	1408.48	1408.48	
10° × 10°	LS-PCS	37765 × 44416	655.47	3979.17	4,634.64	19.71
	LS-WPC	36001 × 36001	0.00	3721.01	3721.01	
14° × 14°	LS-PCS	51664 × 62538	923.21	5960.43	6883.64	17.04
	LS-WPC	50401 × 50401	0.00	5710.69	5710.69	

## Usage Notes

The potential applications of this dataset are as follows: first, it could be used as high-quality input data for global soil erosion assessment, meeting the needs of global soil erosion surveys and promoting erosion topographic analysis and erosion geomorphology research^65^. Second, this dataset could provide a basis for comprehensive evaluations of soil health and other ecosystem service functions^66^. Third, it could help facilitate the evaluation of the economic benefits of land-use planning measures and policies, which could provide a scientific basis for policy-making and land management on a regional or global scale^67^. Finally, this dataset could also be used as a reference in the comparison to other regional soil erosion surveys, global soil erosion surveys, and future soil erosion assessments, as the availability of real data is important for soil erosion models.

While advancements in using relatively high-resolution input data and improved methods have enhanced the quality of the dataset, certain limitations persist. There are certain difficulties in regard to the trade-off between the calculation feasibility and the simulation accuracy in large-scale modelling. The calculation of the LS-factor imposes a spatial scale effect on the input data, which is one of the reasons causing the differences between global-scale estimations (our study) and watershed-scale estimations (other studies). In recognition of this drawback, we offer dedicated software, empowering users to flexibly compute the topographic factor in specific areas. The finer-resolution input data are instrumental in generating more reliable results. With technological advancements, it has become possible to extract LS-factor datasets based on global high-resolution topographic maps.

### Supplementary information

Supplementary File 1-Data link usage instructions

Supplementary Table 1

Supplementary Table 2

Supplementary Table 3

Supplementary Table 4

Supplementary Table 5

Supplementary Table 6

Supplementary Table 7

Supplementary Table 8

Supplementary Table 9

Supplementary Table 10

Supplementary Table 11

Supplementary Table 12

## Data Availability

The LS-WPC method is integrated into LS-TOOL, software that functions as a tool for computing crucial topographic attributes, including the slope steepness, slope steepness factor, slope length, slope length factor, and LS-factor, which play a vital role in the assessment and evaluation of soil erosion. LS-TOOL allows users to flexibly compute the topographic factor in specific regions of interest by simply inputting the desired analysis area. The maximum computable area size depends on the physical memory of the computer. The graphical user interface (GUI) of LS-TOOL is shown in Fig. 13. The areas denoted by the red letters A, B, C, and D are referred to in the text (where details are provided). Area A: Selection of the data type, DEM file, and output file path; Area B: calculation options, including file prefix, models, use of cut-off or not, whether to fill no-data or sink cells, how to fill no-data cells (average or minimum value of the surrounding eight cells), consider channels or not, threshold of the accumulated area, and set the cut-off slope value; Area C: algorithm options, single-flow direction (SFD) or multiple-flow direction (MFD); Area D: selection of which file(s) to save (S: slope steepness; L: slope length, S factor, L factor or ALL). LS-TOOL is available at 10.11888/Terre.tpdc.300613, or contact zhm@nwsuaf.edu.cn.
