# Supplementary material for: A new high-resolution global topographic factor dataset calculated based on SRTM
Source: Sci Data. 2024 Jan 20;11:101. doi: 10.1038/s41597-024-02917-w (PMC10799908; doi:10.1038/s41597-024-02917-w)

Data link usage instructions

Data link:
https://data.tpdc.ac.cn/en/data/d6ce51d1-a94a-4b06-af4c-c24d58e7ec52
or:
https://doi.org/10.11888/Terre.tpdc.300613

(Please wait patiently for a few seconds for the page to load or refresh)

1. enter the repository link.

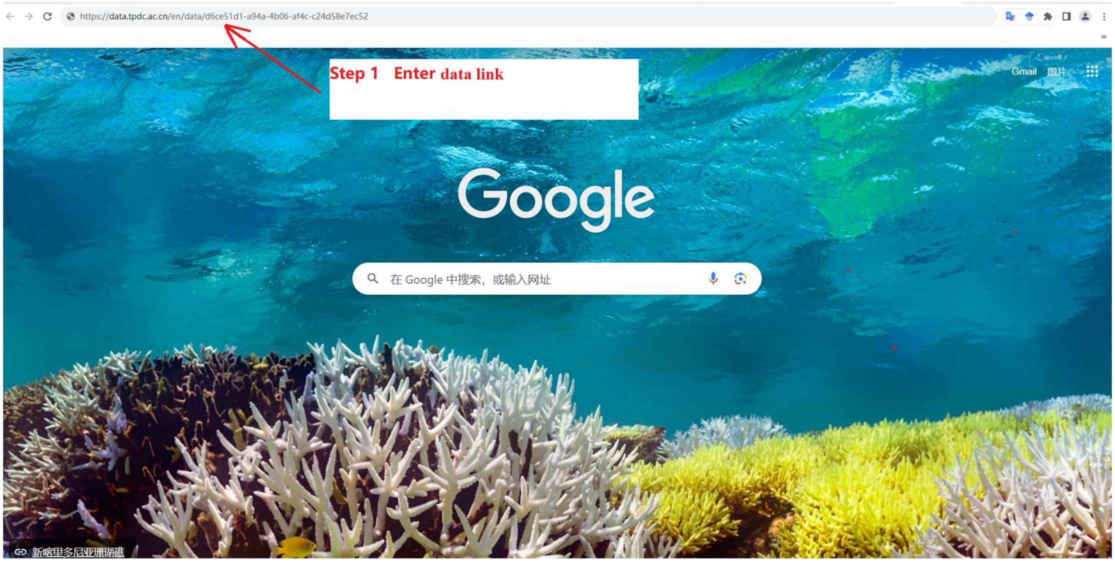

2. click the download button.

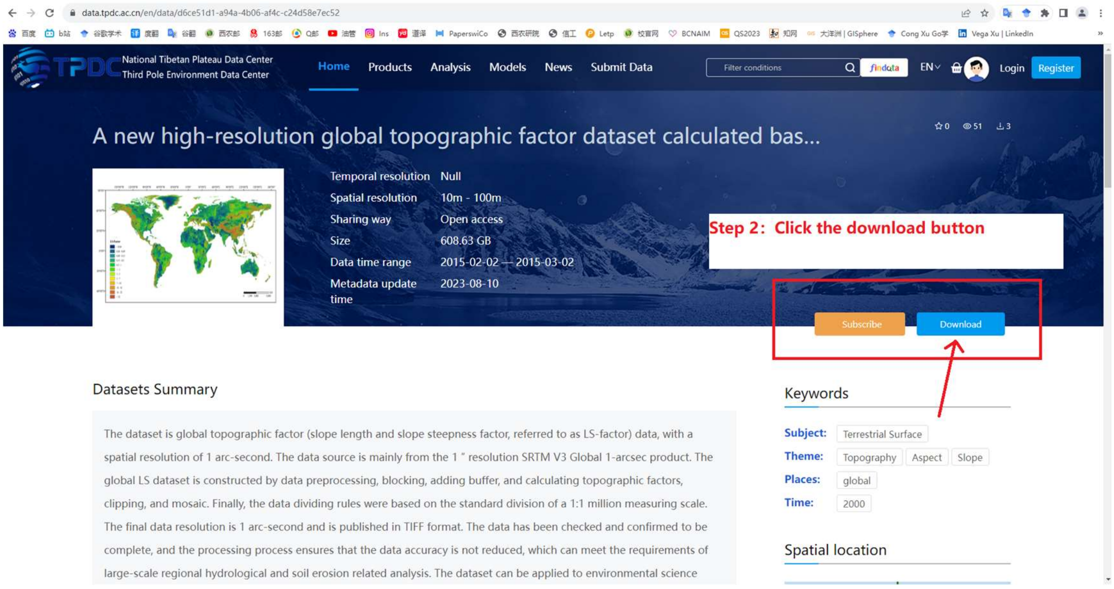

3. due to files larger than 1GB can only be downloaded through FTP server software tools, FTP account is provided. Anyone can anonymously download through this.

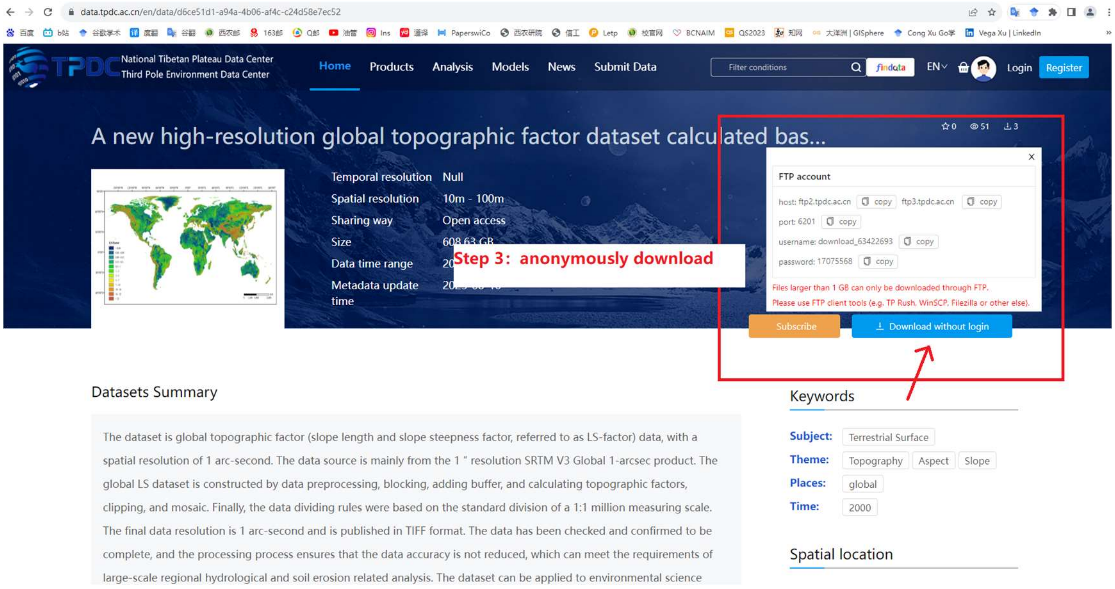

4. Download FTP server software

Download URL: https://www.wftpserver.com/

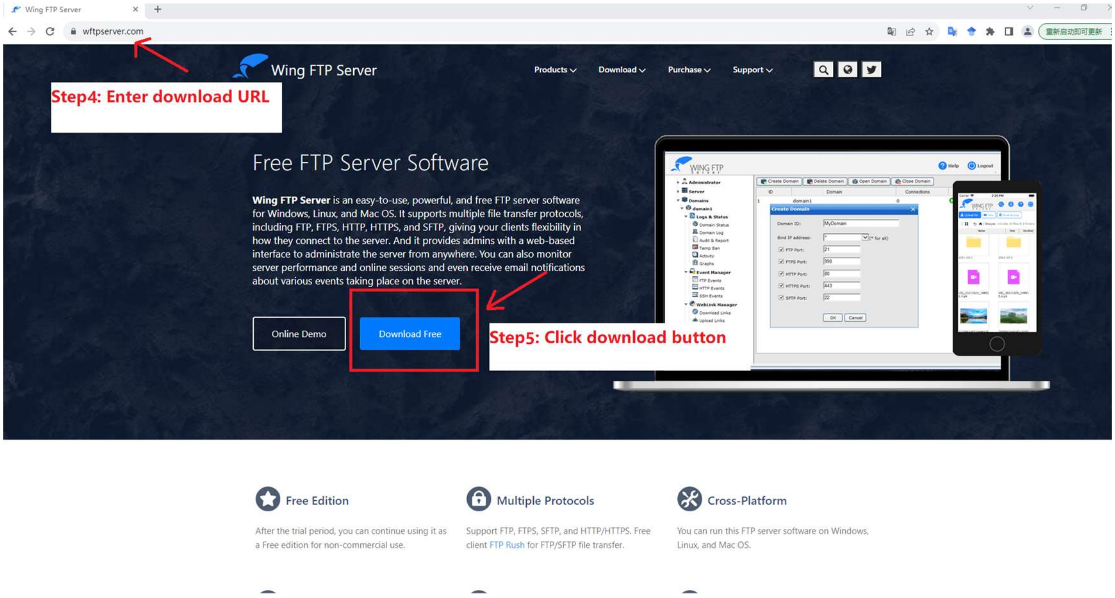

5. Scroll down the page to find FTP Rush v3 and select a version to download based on your computer operating system.

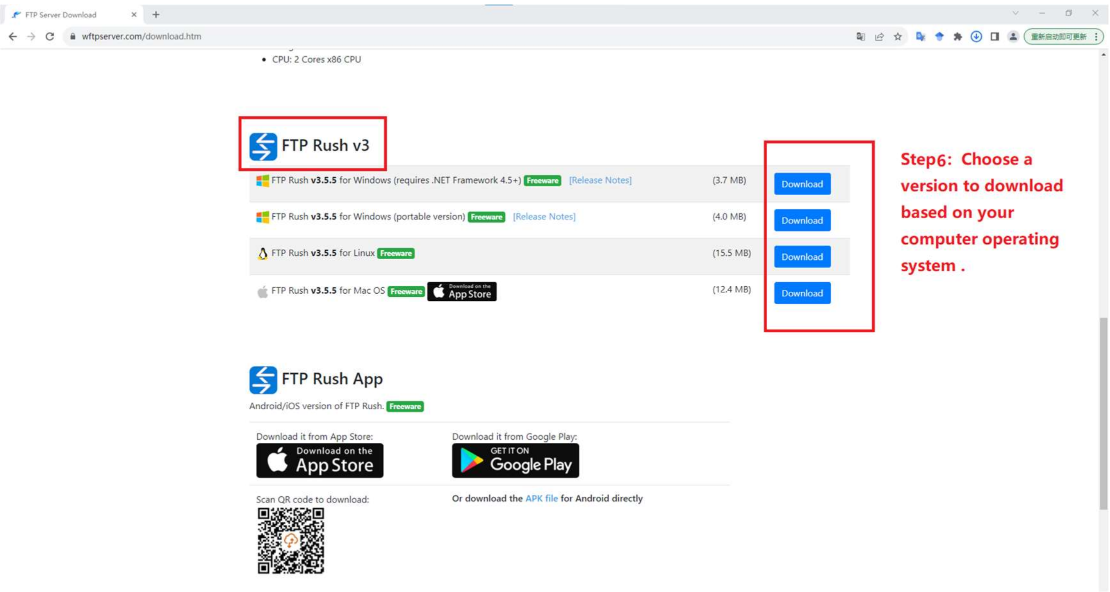

6. After FTP Rush downloads successfully, enter the host, username, password and port provided in step 3, then click the connect button. After the connection is successful, drag the data from the server to the local (As shown in the figure, drag the compressed package on the left to the right)

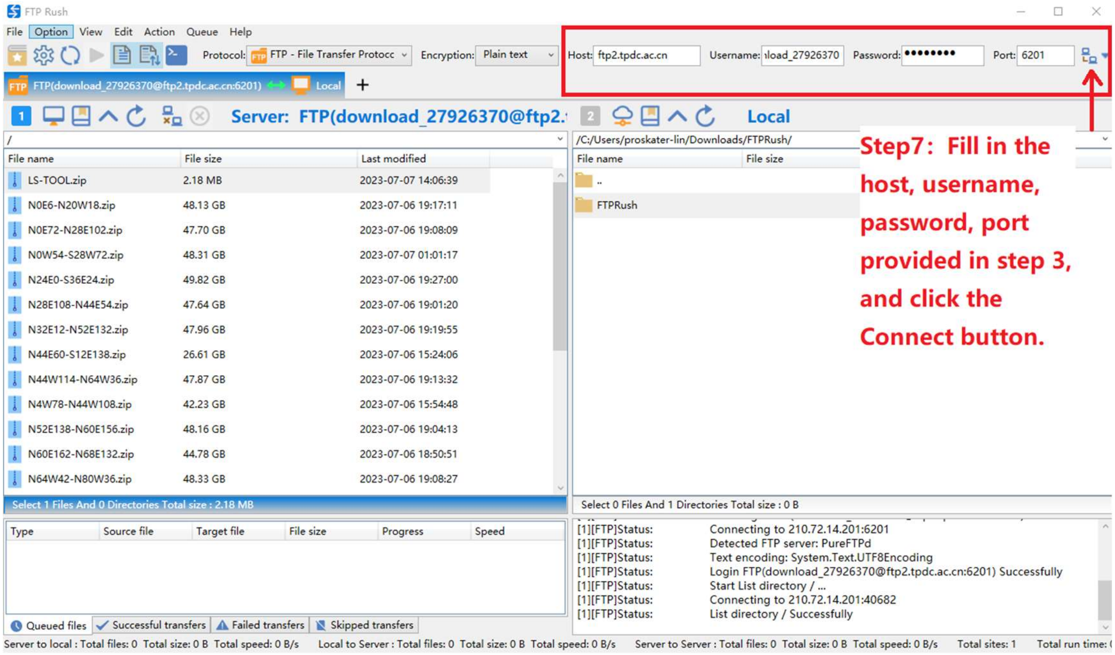

Supplement: Supplementary file 1 — Data link usage instructions [file 41597_2024_2917_MOESM1_ESM.pdf]
